# Dynamic Observation of Autophagy and Transcriptome Profiles in a Mouse Model of Bleomycin-Induced Pulmonary Fibrosis

**DOI:** 10.3389/fmolb.2021.664913

**Published:** 2021-07-29

**Authors:** Yani Wang, Siqi Hu, Lisha Shen, Song Liu, Linyan Wan, Shuhui Yang, Mengjie Hou, Xinlun Tian, Hongbing Zhang, Kai-Feng Xu

**Affiliations:** ^1^ Department of Pulmonary and Critical Care Medicine, State Key Laboratory of Complex Severe and Rare Diseases, Peking Union Medical College Hospital, Chinese Academy of Medical Sciences, Beijing, China; ^2^ Department of Pulmonary and Critical Care Medicine, Subei People’s Hospital of Jiangsu Province, Yangzhou, China; ^3^ Department of Pulmonary and Critical Care Medicine, The First Affiliated Hospital of Zhejiang University School of Medicine, Hangzhou, China; ^4^ Medical Science Center, State Key Laboratory of Complex Severe and Rare Diseases, Peking Union Medical College Hospital, Chinese Academy of Medical Sciences, Beijing, China; ^5^ Department of Physiology, Institutes of Basic Medical Sciences, Peking Union Medical College, Chinese Academy of Medical Sciences, Beijing, China

**Keywords:** idiopathic pulmonary fibrosis, autophagy, RNA-Seq, STEM, bleomycin mouse model

## Abstract

Pulmonary fibrosis is a group of progressive, fibrotic, and fatal lung diseases, and the role of autophagy in pulmonary fibrosis is controversial. In the current research, we dynamically observed a bleomycin-induced pulmonary fibrosis mouse model after 3, 7, 14, 21, and 28 days and investigated the expression of autophagy markers. We found that autophagy markers were not significantly changed on the indicated days in the mouse lung tissue. Then, RNA-Seq was used to analyze the gene expression and associated functions and pathways in fibrotic lung tissue on different days post-bleomycin. In addition, short time series expression miner (STEM) analysis was performed to explore the temporal post-bleomycin gene expression. Through STEM, continually up- or downregulated profiles did not demonstrate the critical role of autophagy in the development of fibrosis. Furthermore, gene ontology (GO) annotations showed that continually upregulated profiles were mainly related to fibrosis synthesis, extracellular space, and inflammation, while enriched pathways were mainly related to the PI3K-Akt signaling pathway, ECM–receptor interactions, and focal adhesion signaling pathway. For continually downregulated profiles, GO annotations mainly involved sarcomere organization, muscle contraction, and muscle fiber development. The enriched KEGG signaling pathways were the cAMP signaling pathway, cGMP-PKG signaling pathway, calcium signaling pathway, and cardiac muscle contraction. Moreover, we analyzed autophagy-related genes’ expression in specific cells from a publicly available database of three human and one animal study of pulmonary fibrosis using single-cell sequencing technology. All results consistently demonstrated no critical role of autophagy in the pathogenesis of pulmonary fibrosis. In summary, autophagy may not critically and consistently change during the development of pulmonary fibrosis at different stages post-bleomycin in a mouse model. These continually up- or downregulated profiles, including gene profiles, and the corresponding functions and pathways may provide mechanistic insights into IPF therapy.

## Introduction

Autophagy plays a pivotal role in maintaining cellular homeostasis and survival (Kundu and Thompson, 2008; Kota et al., 2017). Three types of autophagy have been identified: macroautophagy, microautophagy, and chaperone-mediated autophagy. Among them, macroautophagy is the most prominent form of autophagy and has been extensively studied (Patel et al., 2013). Macroautophagy refers to an evolutionarily conserved pathway that operates *via* lysosome-dependent degradation. The process involves a complicated molecular mechanism that includes more than 30 autophagy-related proteins encoded by autophagy-related genes (Atgs) to ensure that the whole autophagy process proceeds in an orderly manner (Kanthasamy et al., 2006; Komatsu and Ichimura, 2010).

Mounting research has revealed the role of autophagy in pulmonary diseases, such as chronic obstructive pulmonary disease (COPD), pulmonary fibrosis, pulmonary hypertension, cystic fibrosis, and airway remodeling processes (Ryter et al., 2012; Kota et al., 2017). After identifying the role of autophagy in these diseases, several drugs targeting the autophagy process have been developed as therapeutic strategies in the treatment of related diseases and applied in clinical trials (Margaritopoulos et al., 2013). Importantly, whether autophagy represents a target for pulmonary fibrosis therapy is of great interest.

Idiopathic pulmonary fibrosis (IPF) is a kind of progressive and ultimately fatal lung disease that affects more than five million patients worldwide (Chakraborty et al., 2014). Many biomechanical and biochemical factors contribute to the development of IPF, such as aberrant activation of myofibroblasts, injury of the pulmonary epithelium or endothelium, abnormal tissue repair, dysregulation of wound-healing pathways, and accumulation of the extracellular matrix (ECM) (Mora et al., 2017; Richeldi et al., 2017). The median survival of patients diagnosed with IPF is approximately 3 years (Ley et al., 2011). Unfortunately, effective therapies are very limited (Richeldi et al., 2017).

The role of autophagy in IPF is controversial, and decreased or increased expression has been reported in lung tissue obtained from patients with IPF (Patel et al., 2012; Kota et al., 2017). Such inconsistent results were also noted in animal studies with a bleomycin-induced pulmonary fibrosis model in mice (Gui et al., 2015; Zhao et al., 2020a).

Intriguingly, our previous research also found compromised autophagy in mice with an aberrant activation of mTOR in alveolar epithelial cells, which had the potential to develop into more severe pulmonary fibrosis. However, enhancement of autophagy *via* blocking mTOR with rapamycin protected mice from pulmonary fibrosis (Gui et al., 2015). To explore the relationship between autophagy and pulmonary fibrosis, we explored the dynamic changes in autophagy in different phases of a bleomycin-induced mouse fibrosis model. Additionally, we also identified the differentially expressed genes and signaling pathways in different stages of bleomycin-induced mouse fibrosis by transcriptomic sequencing.

## Material and Methods

### Animal Models

C57BL/6J male mice were purchased from Vital River Lab Animal Technology (Beijing, China). 6- to 8-week-old mice weighing 20–25 g were randomly separated into cages, each with five mice. Mice were kept in cages for at least 1 week so that they could adapt to the new environment. For bleomycin-induced pulmonary fibrosis, mice were anesthetized with 0.6% sodium pentobarbital, followed by intratracheal injections of bleomycin (2.5 mg/kg) (Nippon Kayaku Co., Ltd., Tokyo, Japan) or sterile saline, as previously described (Gui et al., 2015). Mice were euthanized at days 3, 7, 14, 21, and 28 after the intratracheal injection of bleomycin or saline. Then lung tissue was collected after perfusion with sterile saline from the right ventricle. The protocol of this study was approved by the Animal Ethics Committee of Peking Union Medical College (XHDW-2017-010).

### Histology and Autophagy Evaluation

Lung tissue was collected, fixed, and embedded in paraffin for hematoxylin–eosin (H&E) staining and Masson trichrome staining. Sections (5 μm thick) were also used for staining of α-SMA. Immunofluorescent staining of LC3B dots was performed using Alexa Fluor 488 conjugated secondary IgG. Immunofluorescence of LC3B dots was visualized using an Olympus confocal microscope. Transmission electron microscopy (TEM) was used to evaluate autophagosomes in the lung tissue. For Western blotting, antibodies against SQSTM1 (p62) (Cell Signaling Technology, Danvers, MA), LC3B (Abcam, Cambridge, MA), α-SMA, and β-actin (Cell Signaling Technology, Danvers, MA) were used. Detailed experimental procedures are described in the Supplementary Material.

### Total RNA Extraction, cDNA Library Construction, and RNA-Seq

6- to 8-week-old C57BL/6J male mice weighing 20–25 g were anesthetized with 0.6% sodium pentobarbital, followed by intratracheal injections of bleomycin (2.5 mg/kg). Mice were euthanized at the indicated day post-bleomycin. For RNA-Seq, the mouse lung tissue was rinsed in PBS to remove blood, and about 30 mg of mice lung tissue was collected to extract the total RNA using TRIzol (Life Technologies, MA, United States) reagent. Then RNA integrity and quantity were controlled and measured. The whole RNA was then disrupted into several fragments for the synthesis of cDNA. The quality of the cDNA library was tested using an Agilent 2,100 bioanalyzer. Finally, RNA sequencing was performed on an Illumina HiSeqTM 2,500 (service supported by OE Biotech Co., Ltd., Shanghai, China) platform, generating 150-bp paired-end reads. 49 million raw data of each sample were generated.

### Bioinformatics Analysis

The threshold of significantly differentially expressed genes was set at a *p* value of <0.05 and a fold change of >2 or <0.5. Gene ontology (GO) terms in the biological process (BP), cellular component (CC), and molecular function (MF) categories were analyzed using the Database for Annotation, Visualization and Integrated Discovery (DAVID 6.8; https://david.ncifcrf.gov/) (Dennis et al., 2003). The Kyoto Encyclopedia of Genes and Genomes (KEGG) pathway annotation was conducted using KEGG Orthology-Based Annotation System (KOBAS) online tools (http://www.genome.jp/kegg) (Kanehisa et al., 2008).

### Quantitative RT-PCR

Total lung tissue RNA was extracted using TRIzol (Life Technologies, MA, United States) reagent according to the manufacturer’s instructions. 1 μg RNA was used as the template to synthesize the first-strand cDNA using a PrimeScript^TM^ RT Master Mix Kit (Takara, Shiga, Japan). Then cDNA was diluted 20 times and used as the template for real-time PCR using a TB Green Premix Ex Taq^TM^ II Kit (Takara, Shiga, Japan). The total reaction system was 20 μL, and the gene amplification process was performed using an Applied Biosystems 7,500 Fast Real-Time PCR System (Life Technologies). The RNA expression of target genes was calculated using the 2^−ΔΔCt^ method on the basis of the housekeeping gene β-actin. Every sample was performed three times in triplicate. The primers of the target genes are listed in Supplementary Table S1.

### Autophagy-Related Gene Expression Validation With Public Data Source

To further validate autophagy in specific cells, such as airway epithelial cells and fibroblasts in lung tissue obtained from IPF patients and animal models, gene expression data were downloaded from the GEO profiles of three scRNA-Seq studies (GSE136831, GSE135893, and GSE86618) for human IPF lungs and one bleomycin-induced mouse pulmonary fibrosis model (GSE141259) (Xu et al., 2016; Adams et al., 2020; Habermann et al., 2020; Strunz et al., 2020). The Seurat V4 (Hao et al., 2020) package was used to analyze the data. For each dataset, the mtx file, cellbarcode file, and geneID files were loaded into R software, and the cell types and group information were acquired from the metatable. Then, the autophagy-related gene expression was extracted and compared between IPF or fibrosis and the control group.

### Statistical Analysis

The data are shown as the mean ± standard error. The differences between two groups were compared using Student’s *t*-test. One-way ANOVA was used to compare the differences between more than two groups. Statistical differences were identified at *p* < 0.05.

## Results

### Autophagy Is Not Consistently Changed in the Pulmonary Fibrosis Mouse Model

Male C57BL/6J mice weighing 20–25 g were used to establish a bleomycin-induced (2.5 mg/kg; intratracheally) pulmonary fibrosis model. Five time points were used: the baseline and 3, 7, 14, 21, and 28 days after bleomycin treatment. A detailed experiment on this model is described in the Supplementary Material.

**FIGURE 1:**
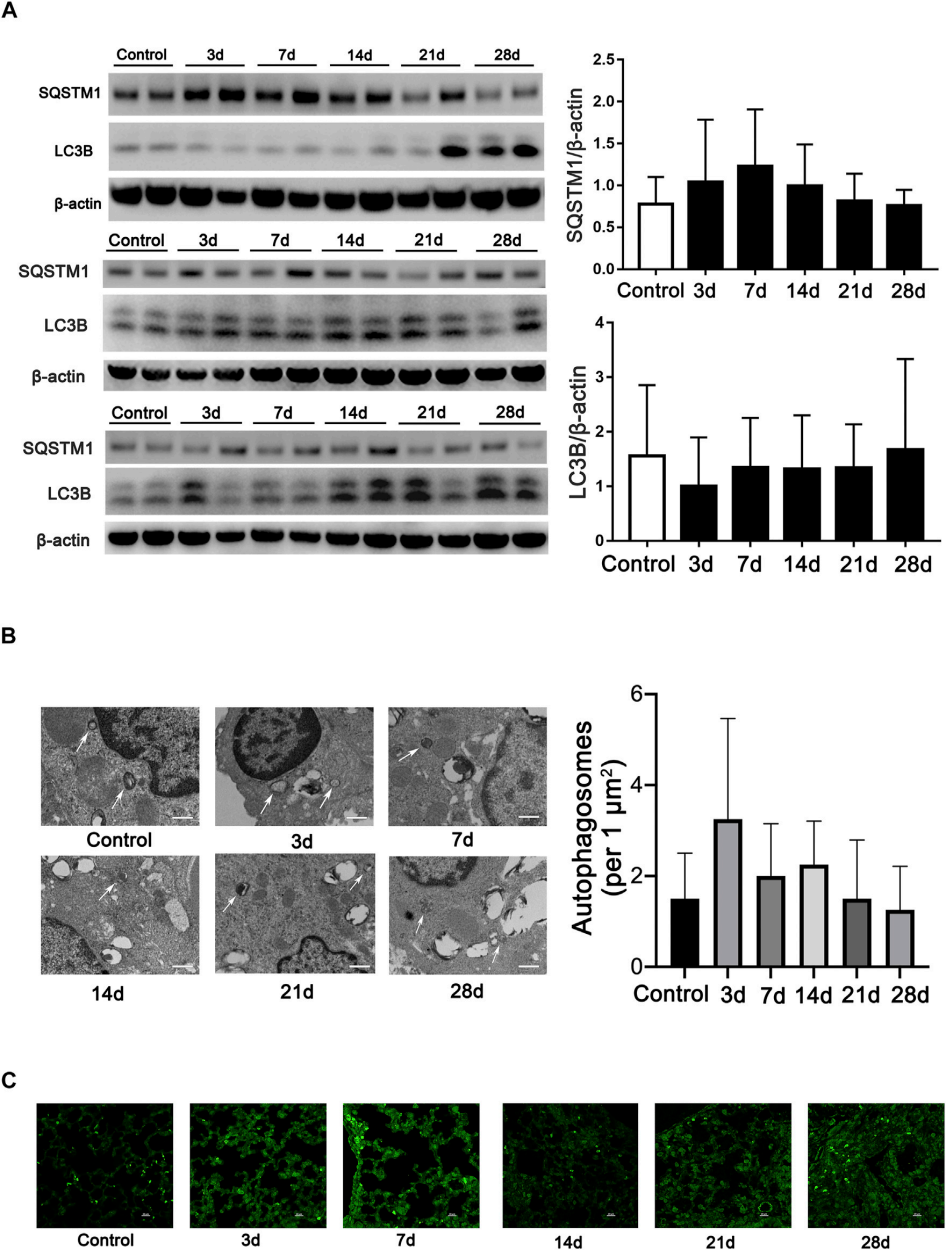
Autophagy is not continually induced in pulmonary fibrosis mouse models. **(A)** Immunoblots and semiquantification (*n* = 6) for SQSTM1 (P62) and LC3B in mouse lung tissue on the indicated days post-bleomycin. Mouse lung tissue was collected and subjected to Western blotting using the indicated antibodies. **(B)** Representative transmission electron microscopy images of autophagosomes and semiquantification of autophagosomes (*n* = 6) in pulmonary alveolar epithelial cells of post-bleomycin mice on the indicated days. White arrows represent autophagosomes. Scale bar: 1 μm. **(C)** Representative fluorescence microphotographs of LC3B dots in mouse lung tissue. Scale bar: 20 μm.

Microtubule-associated protein 1A/1B-light chain 3 (LC3B), an essential protein for autophagosome formation, was used to monitor autophagy in cells. The semiquantification of LC3B calculated in all mice indicates no difference among the groups. Another marker of autophagy, sequestosome 1 (SQSTM1/p62), which exhibited a negative relationship with the activation of autophagy, also exhibits no difference in all the mice among the groups (Figure 1A). We then monitored the formation of autophagosomes using TEM analysis. However, the results showed that few autophagosomes were stimulated during fibrosis induced by bleomycin (Figure 1B). In addition, we also observed LC3-positive dots by immunofluorescent staining in every mouse tissue slice post-bleomycin on the indicated day. The results showed that LC3-positive puncta between the groups did not significantly increase in the mice treated with bleomycin (Figure 1C). Taken together, autophagy did not show consistently increased or decreased expression in different stages of the pulmonary fibrosis mouse model induced by bleomycin.

### Gene Ontology Enrichment Analysis of Differentially Expressed Genes at Different Stages of Pulmonary Fibrosis

RNA-Seq was performed to investigate differentially expressed genes (DEGs) at different stages of pulmonary fibrosis. After principal component analysis (PCA) (Supplementary Figure S2), numerous DEGs were found at different time points in the animal model (Supplementary Figure S3).

**FIGURE 2:**
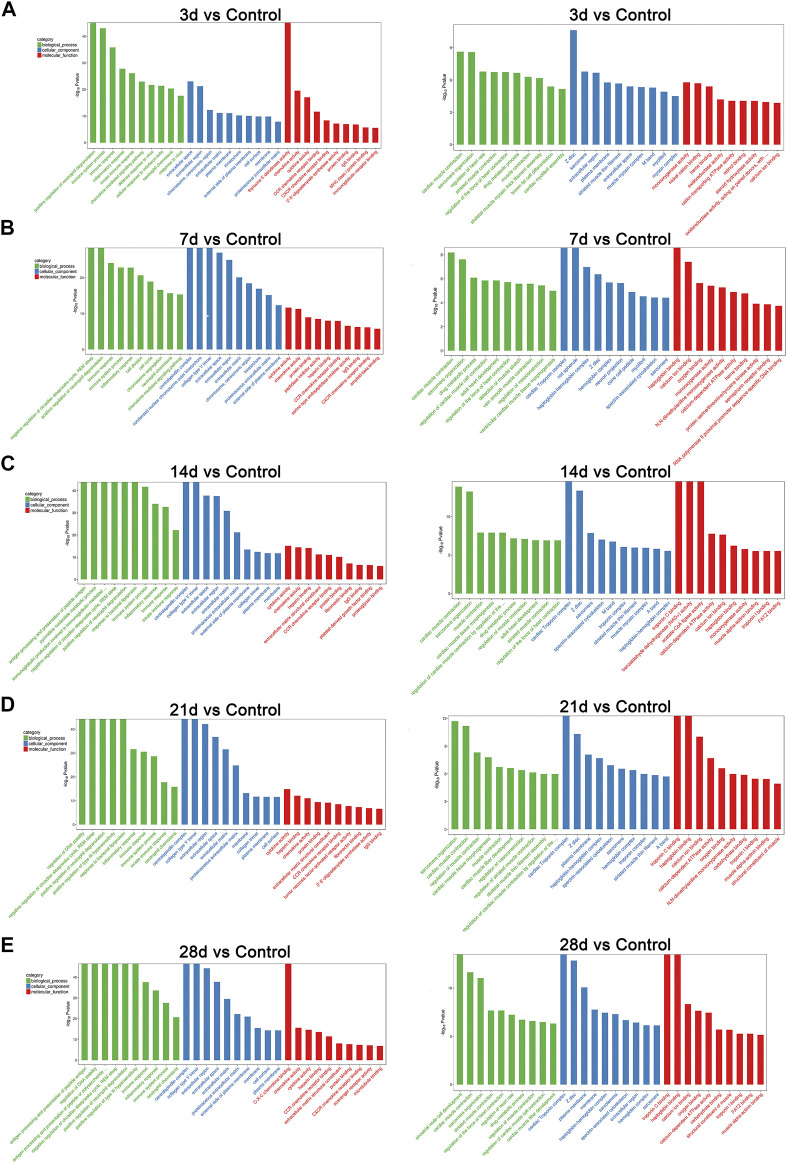
GO analysis of differentially expressed genes at different stages of post-bleomycin mice. The top 30 GO annotations of differentially expressed genes between the group of mice treated with bleomycin and the control are shown. **(A)** Third day post-bleomycin vs. the control. **(B)** Seventh day post-bleomycin vs. the control. **(C)** Fourteenth day post-bleomycin vs. the control. **(D)** Twenty-first day post-bleomycin vs. the control. **(E)** Twenty-eighth day post-bleomycin vs. the control. **(A–E)** GO analysis describing the possible functions of differentially expressed genes through the biological process, cellular component, and molecular function categories. Green indicates the biological process, blue indicates the cellular component, and red indicates the molecular function. Left panel, upregulation; right panel, downregulation.

To determine the related functions of the DEGs involved, a gene ontology (GO) enrichment analysis was performed. GO terms interpret possible functions of DEGs through the biological process (BP), cellular component (CC), and molecular function (MF) categories. We compared the functions of many DEGs that were involved on different days between the bleomycin group and the control group. Among those annotations, on the third day post-bleomycin, the biological processes of the lung tissue, such as neutrophil degranulation, immune system–related processes, and inflammatory responses, were significantly promoted. Additionally, these biological processes were stimulated on the seventh, 14th, 21st, and 28th days post-bleomycin. However, sarcomere organization, muscle contraction–related processes, and development were significantly downregulated. For annotations of cellular components, bleomycin facilitated extracellular components, collagen trimers, and membranes. Annotations of sarcomeres and extracellular space– and muscle-related structures were downregulated. For molecular function terms, annotations for chemokines, cytokines, and receptor activation were highly promoted, while functions of oxygen binding, troponin C binding, and calcium ion binding were downregulated. As depicted in Figure 2, the top 30 annotations classified into the biological process, cellular component, and molecular function categories are displayed. Taken together, bleomycin has a great effect on mouse immune system processes (GO:0002376), muscle systems (GO:0003012), extracellular regions (GO:0005576), and contractile fibers (GO:0043292).

### Kyoto Encyclopedia of Genes and Genomes Signaling Pathway Analysis

**FIGURE 3:**
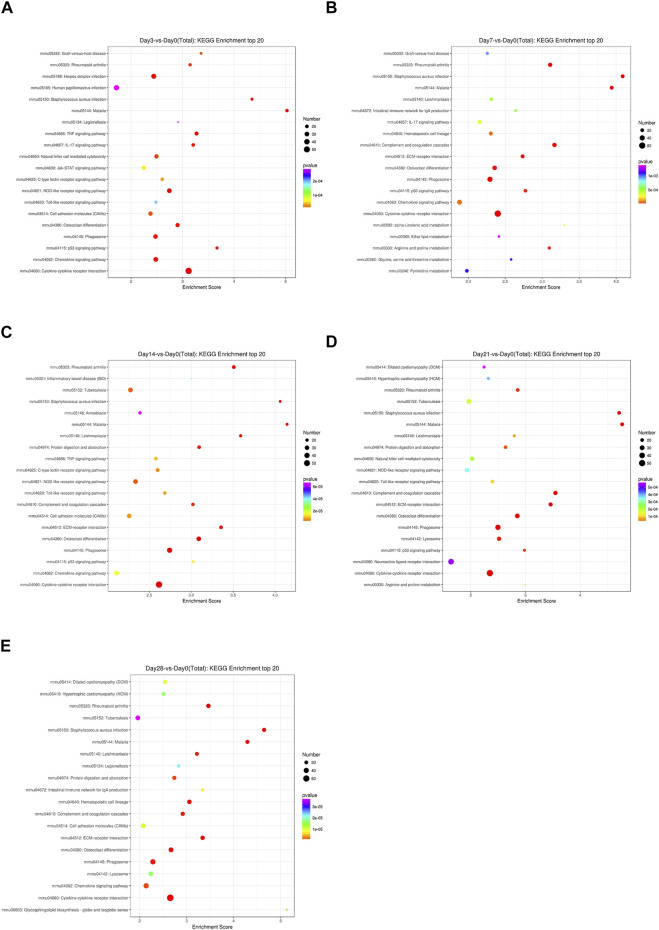
KEGG signaling pathways of differentially expressed genes at different stages in bleomycin-induced fibrosis models. The top 20 KEGG pathways of differentially expressed genes between the group of mice treated with bleomycin and the control are shown. **(A)** At day 3 post-bleomycin vs. the control. **(B)** At day 7 post-bleomycin vs. the control. **(C)** At day 14 post-bleomycin vs. the control. **(D)** At day 21 post-bleomycin vs. the control. **(E)** At day 28 post-bleomycin vs. the control. **(A–E)** X-axis indicates the enrichment scores. The size of the bubble indicates the number of differentially expressed genes enriched in the indicated pathway.

To identify the significantly expressed signaling pathways in which the DEGs were involved, a KEGG enrichment analysis was performed on the indicated day post-bleomycin. In comparison with the control, on the third day post-bleomycin, cytokine–cytokine receptor interactions, the NOD-like receptor signaling pathway, and the chemokine signaling pathway were upregulated, while the PPAR signaling pathway, adrenergic signaling in cardiomyocytes, and the calcium signaling pathway were mainly enriched and significantly downregulated (Figure 3A). At day 7, a total of 50 significantly upregulated DEGs were involved in cytokine–cytokine receptor interactions and other activated pathways involved in phagosome and chemokine signaling pathways. Meanwhile, adrenergic signaling in cardiomyocytes, neuroactive ligand–receptor interactions, and calcium signaling pathways were downregulated (Figure 3B). Similarly, cytokine–cytokine receptor interactions, osteoclast differentiation, the chemokine signaling pathway, and phagosomes were also upregulated at day 14, while adrenergic signaling in cardiomyocytes, dilated cardiomyopathy (DCM), and cAMP signaling were downregulated (Figure 3C). On the 21st day post-bleomycin, significantly upregulated DEGs were abundant in cytokine–cytokine receptor interactions and the lysosome and osteoclast differentiation signaling pathways, and downregulated DEGs were mainly enriched in neuroactive ligand–receptor interaction, cardiac muscle contraction, and adrenergic signaling in the cardiomyocyte signaling pathway (Figure 3D). At day 28, cytokine–cytokine receptor interactions, *Staphylococcus aureus* infection, rheumatoid arthritis, and cell adhesion molecule–related signals were significantly upregulated. For the downregulated signals, the DEGs were mainly abundant in the dilated cardiomyopathy (DCM)-, cardiac muscle contraction–, and hypertrophic cardiomyopathy (HCM)-related signaling pathways (Figure 3E). Taken together, the KEGG pathway analysis results identified the upregulation of cytokine–cytokine receptor interactions and the chemokine signaling pathway and the downregulation of the cardiomyopathy-related signaling pathway as the consistent characteristics of pulmonary fibrosis.

### Short Time Series Expression Miner Analysis of Differentially Expressed Genes

**FIGURE 4:**
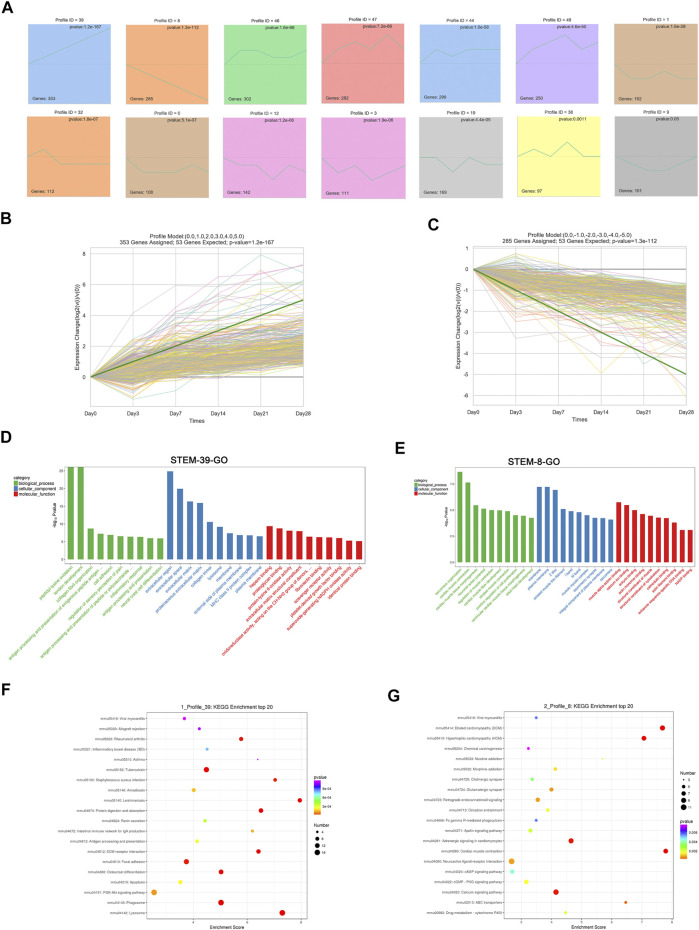
Short time series expression miner (STEM) analysis of DEGs at different stages. **(A)** STEM analysis identified 14 significantly expressed profiles with a *p* value of <0.05. The numbers of genes in each profile are shown in the bottom left corner, and the *p* value of each corner is shown in the top right corner. **(B)** Expression patterns of Profile 39. **(C)** Expression patterns of Profile 8. **(D)** GO analysis of Profile 39. **(E)** GO analysis of Profile 8. **(D–E)** GO analysis of profiles using the biological process, cellular component, and molecular function categories. Green indicates the biological process, blue indicates the cellular component, and red indicates the molecular function. **(F)** Top 20 KEGG pathways of Profile 39. **(G)** Top 20 KEGG pathways of Profile 8. **(F–G)** X-axis indicates the enrichment scores. The size of the bubble indicates the number of genes enriched in the indicated pathway.

To address the gene expression patterns in pulmonary fibrosis at different stages post-bleomycin, STEM analysis was performed. STEM analysis can cluster, compare, and visualize gene expression data for short time series and their expression trends at different times to help identify the significantly expressed genes. As a result, 50 profiles were determined. Among them, 13 profiles were significantly expressed with a *p* value of <0.05. As depicted in Figure 4A, the green line in each profile represents the expression pattern of the genes at different times. The total number of genes in each profile cluster is presented on the bottom left. Of note, among all profiles determined, we found that Profile 39 involved 353 genes that were continually upregulated and Profile 8 included 285 genes that were continuously downregulated (Figures 4B,C). Other profiles showed biphasic expression patterns. To identify the major functions and signaling pathways of the continuously upregulated and downregulated genes, GO annotations and KEGG pathway analysis were conducted.

According to the GO annotations, the upregulated biological process of genes in Profile 39 was mainly enriched in collagen fibril organization, cell adhesion, and inflammatory response. Cellular component annotations for the extracellular region, extracellular space, and extracellular matrix were mainly enriched. For molecular functions, protein binding, such as heparin, plate-derived growth factor binding, and fibronectin binding, was continually upregulated (Figure 4D). DEGs encoding fibrillar collagen (I, Ⅳ, Ⅴ, Ⅶ, etc.), ADAM metallopeptidase (Adamts1, Adam12, Adamts7, etc.) procollagen processing enzymes (ADAMTS), and the extracellular matrix (Ctsd, Mmp2, Apoe, etc.) were mainly involved (Supplementary Excel 2). For the consistently downregulated Profile 8, annotations for biological processes referred to muscle contraction, sarcomere organization, and muscle fiber development. Cellular component terms, such as the membrane, muscle myosin complex, and striated muscle thin filament, were downregulated. For the molecular function, continually downregulated annotations included muscle alpha-actinin binding, calcium ion binding, and the structural constituent of the cytoskeleton (Figure 4E). An analysis of related DEGs implicated genes involving muscle contraction (Actc1, Tnnc1, Casq2, etc.), sarcomeres (Tnnt2, Lmod2, Ttn, etc.), and membranes (Slc47a1, Lepr, Cyp3a13, etc.) (Supplementary Excel 3).

To determine the regulated pathways of the continually upregulated pattern Profile 39 and the continually downregulated pattern Profile 8, a KEGG pathway analysis of these two patterns was performed. As shown in Figure 4F, pathways such as PI3K-Akt signaling, phagosome, ECM–receptor interaction, and lysosome were significantly upregulated and related genes such as Col1a1, Col1a2, Col4a1, Ctsa, Ctsb, Ctsd, Itga11, and Itgb6 were involved (Supplementary Excel 4). For the continually downregulated pattern Profile 8, annotations were mainly enriched in cAMP signaling, PKG signaling, calcium signaling, and cardiac muscle contraction (Figure 4G). Genes such as Actc1, Adcy8, Dmd, Slc8a3, Adrb2, and Myh6 were enriched in these pathways (Supplementary Excel 5).

**FIGURE 5:**
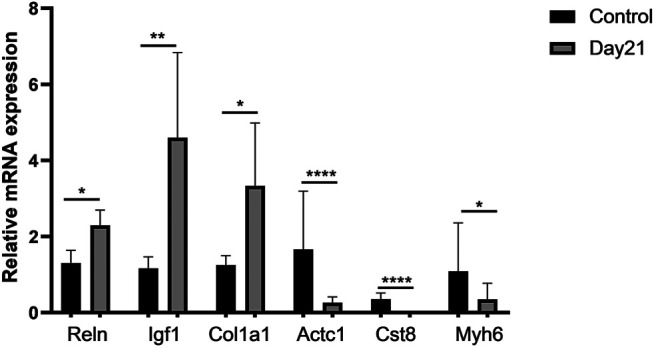
Validation of representative genes in differentially expressed pathways. A total of six differentially expressed genes (DEGs) involving three upregulated DEGs, Reln, Igf1, and Col1a1, and three downregulated DEGs, Actc1, Cst8, and Myh6, were validated by qRT-PCR. Total RNA of mouse lung tissue was extracted using TRIzol, and then qRT-PCR was performed after cDNA was constructed. Every sample was performed three times in triplicate. Each error bar indicates the mean ± SD of eight mice per group. *p < 0.05, **p < 0.01, ****p < 0.0001.

We selected several genes from differentially expressed pathways for validation, including Reln, Igf1, Col1a1, Actc1, Cst8, and Myh6, and found that the results were consistent with the predicted changes of pathways in RNA-Seq (Figure 5).

### Expression of Autophagy-Related Genes

**FIGURE 6:**
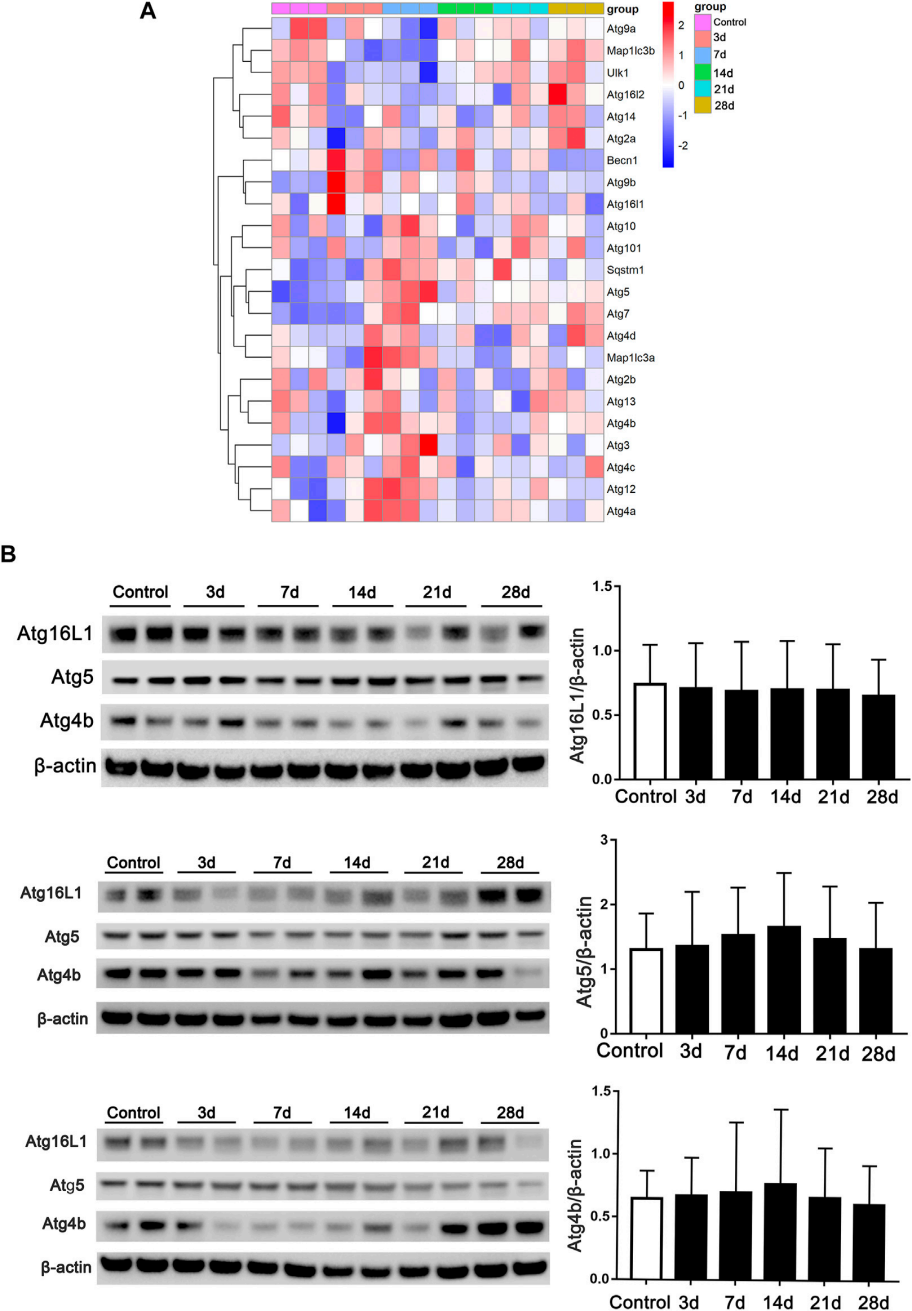
Expression of autophagy-related genes in RNA-Seq at different stages of fibrosis. **(A)** Expression of autophagy-related genes obtained from RNA-Seq at different stages of fibrosis. **(B)** Validation of autophagy-related genes and semiquantification (*n* = 6) by Western blot. Mouse lung tissue was collected and subjected to Western blotting using the indicated antibodies.

Autophagy-related genes at the post-bleomycin transcriptomic level in different stages were analyzed. We focused on the critical genes that play a critical role in the process of autophagy or in the formation of autophagosomes using RNA-Seq. As shown in Figure 6A, the heatmap revealed the expression pattern of the genes in different stages. However, autophagy-related genes did not show continually significant differential expression at the different stages post-bleomycin. We also chose several genes for validation by immunoblotting and compared the results with those of RNA-Seq (Figure 6B). The results were consistent with the RNA-Seq results, which showed that these genes did not continually regulate pulmonary fibrosis. Taken together, we postulated that autophagy was not consistently increased or decreased in the development of pulmonary fibrosis at different stages of fibrosis induced by bleomycin.

### Autophagy-Related Gene in Single-Cell Sequencing Studies

No consistent changes of autophagy were found in our experiment. We wish to know whether autophagy is changed in specific cells in the lung, especially in airway epithelial cells and fibroblasts. We analyzed data from several studies of single-cell sequencing which allow us to know the information of autophagy in different subtypes of cells (Xu et al., 2016; Adams et al., 2020; Habermann et al., 2020; Strunz et al., 2020). After analysis of the database from both the human IPF studies and the animal dynamic observation study in mice with the bleomycin-induced fibrosis model, we found that there were no consistent changes of autophagy expression in lung samples from both human and animal studies.

**FIGURE 7:**
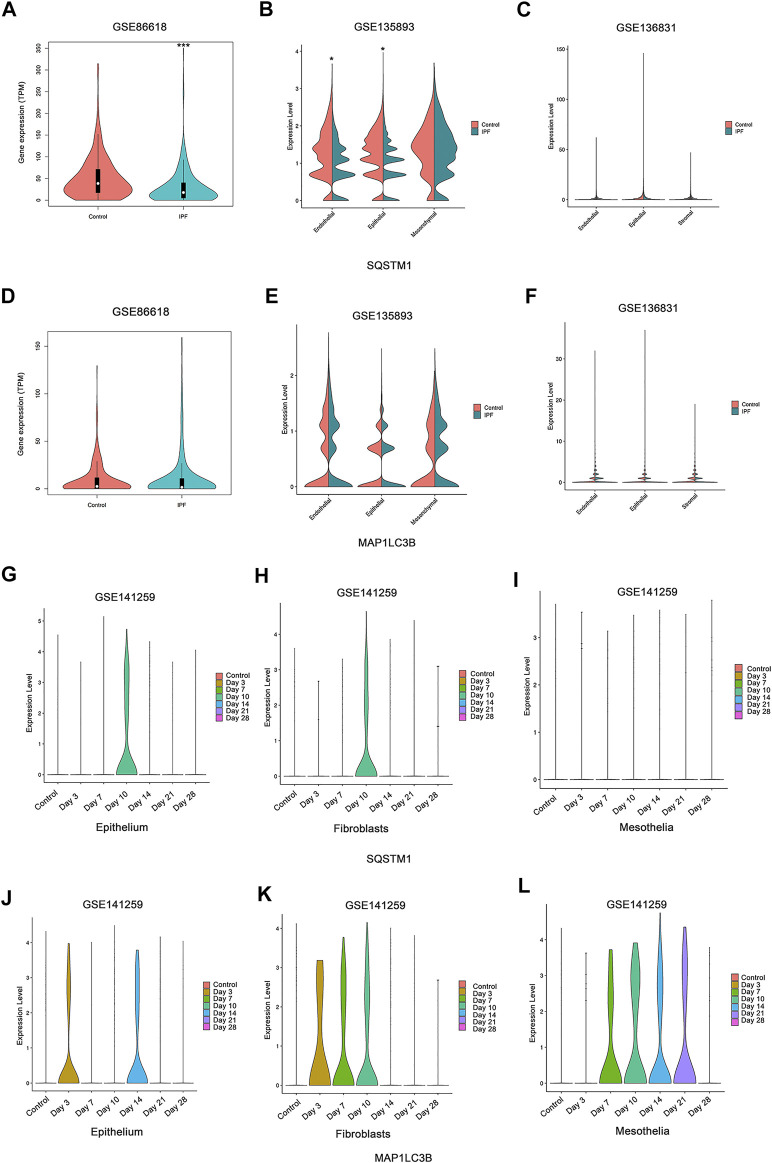
Expression of autophagy in single-cell sequencing studies. **(A,D)** Expressions of SQSTM1 and MAP1LC3B were analyzed among idiopathic pulmonary fibrosis (IPF) patients and the health from NCBI Gene Expression Omnibus (GEO) dataset GSE86618. **(B,E)** Expressions of SQSTM1 and MAP1LC3B were analyzed among IPF patients and the health from GEO dataset GSE135893. **(C,F)** Expressions of SQSTM1 and MAP1LC3B were analyzed among IPF patients and the health from GEO dataset GSE136831. **(A–C)** Expression of SQSTM1. **(D–F)** Expression of MAP1LC3B, *p < 0.05, ***p < 0.001. **(G–L)** Expressions of SQSTM1 and MAP1LC3B were analyzed within the indicated cell subtype in pulmonary fibrosis mouse models induced by bleomycin at different stages. Data was from NCBI GEO dataset GSE141259. **(G–I)** Expression of SQSTM1. **(J–L)** Expression of MAP1LC3B.

For most of the autophagy-related genes, the expression levels were almost undetectable within most of the cells (data not shown). For the detected genes, SQSTM1 shows slight downregulation in the epithelial cells in two of three studies on human IPF patients (Figures 7A,B), and no other stable dysregulation of autophagy-related genes involving Map1LC3B (Figures 7D–F) was detected. In the research on mouse fibrosis induced by bleomycin using sc-RNA, SQSTM1 was only expressed at day 10 post-bleomycin in the mouse epithelium and fibroblasts (Figures 7G,H), while no expression was detected on other days and no expression was detected at the mesothelia (Figures 7G–I). Meanwhile, Map1LC3B was upregulated from day 3 post-bleomycin in the mice pulmonary epithelium and fibroblasts while exhibiting an increased expression in the mesothelia at day 7 post-bleomycin (Figure 7L). Although it was expressed between day 7 and day 21 post-bleomycin in mesothelia, Map1LC3B was not expressed at day 21 and 28 post-bleomycin in epithelium and fibroblasts. Of note, besides Map1LC3B and SQSTM1, other autophagy-related genes were also not detectable (data not shown).

## Discussion

Intratracheal instillation of bleomycin is commonly used for studying the pathogenesis of pulmonary fibrosis because of the strong fibrosis process response. This model is based on the initiation of the inflammatory process followed by the conversion to fibrosis. At the beginning of the first 7 days post-bleomycin, a strong inflammation was induced in response to the lung injury. The fibrosis formation can be observed at day 14 and reached its plateau period between day 21 and day 28 in single-dose intratracheal administration of bleomycin, followed by the resolution of the fibrosis within 6 weeks (Moore and Hogaboam, 2008; Degryse et al., 2010). Hence, the mice treated with bleomycin were divided into five groups to better explore the role of autophagy in the different stages as well as differentially expressed genes and their related functions in the development of pulmonary fibrosis.

To observe the dynamic changes in autophagy markers in the development of a mouse model of bleomycin-induced pulmonary fibrosis, lung samples were collected at the baseline and after 3, 7, 14, 21, and 28 days post-bleomycin. Results from immunoblotting have showed that autophagy marker LC3B and SQSTM1/p62 expressed no difference in all the mice among the groups. In addition, other autophagy-related genes also showed little change in the whole lung tissue of fibrosis. Given that the formation of autophagosomes is the gold standard of autophagy, we then observed autophagosomes by TEM. Previous research studies have observed the autophagosomes in epithelial cells. In a study, the numbers of autophagosomes between IPF patients and the healthy lung had no significance, and autophagy was not induced in IPF patients (Patel et al., 2012). Cabrera S. et al. observed autophagosomes in pulmonary epithelial cells, which they thought that the autophagy gene was highly abundant in the pulmonary epithelial cells (Cabrera et al., 2015). Our observation did not show a significant difference in the number of autophagosomes in the epithelium by TEM. Based on our study, we cautiously concluded that autophagy is probably not critically involved in the development of pulmonary fibrosis.

Moreover, the RNA-Seq results indicated that autophagy-related genes expressed in different stages of fibrosis did not exhibit a significant continuous increase or decrease in expression. Validation by immunoblotting was consistent with the RNA-Seq results. Many genes are involved in the process of macroautophagy and exert a critical role. For instance, ATG4b was reported to be another important protein regulating the formation of autophagosomes by modifying LC3 (Satoo et al., 2009). ATG5 is responsible for autophagic death (Klionsky et al., 2003; Pyo et al., 2005). ATG16 L1, as a binding protein with ATG5, also plays a pivotal role in the process of autophagy (Mizushima et al., 2003). However, we did not find evidence supporting significant increases or decreases in autophagy during the development of fibrosis.

In the literature, autophagy has been reported to be at the low basal level in a mouse model of pulmonary fibrosis induced by bleomycin (Chitra et al., 2015). In human samples from IPF patients, autophagy was not induced in some studies (Patel et al., 2012; Araya et al., 2013). Based on controversial reports from the literature and the current research reported here, we postulate that autophagy may not exert a critical role in the process of fibrosis.

Autophagy is likely not consistently changed, overall, among the total lung tissue samples. Thus, we sought to determine whether variations in autophagy occur in specific cells, such as airway epithelial cells and fibroblasts/myofibroblasts. Recently, single-cell sequencing studies have provided some answers. In two research studies on human IPF, decreased expression of SQSTM1/p62 was shown. Meanwhile, in all three research studies, expression of LC3B and other autophagy-related genes showed no difference among the cell subtype and the disease. As for the results from a research study using bleomycin-induced mouse models, the expression of autophagy seems increased in a short period at the inflammatory stage based on the characteristics of mouse models induced by bleomycin which are dependent on the development of inflammation, while showing little change in the fibrosis plateau period that usually occurs between day 21 and day 28 (Moeller et al., 2008) (Figure 7).

To further understand the mechanisms of pulmonary fibrosis, a dynamic transcriptome profile provided interesting findings. Numerous genes were either upregulated or downregulated in the model of bleomycin-induced pulmonary fibrosis. DEGs in different stages of fibrosis were analyzed. GO terms explained the functions of these DEGs based on the molecular function, cellular component, and biological process categories. We found that in the biological process category, positive regulation of neutrophil degranulation was significantly upregulated at days 3 and 7 post-bleomycin, and the DEGs Ptafr, Cd177, Itgam, and Itgb2 were mainly involved in this process. In bleomycin-induced murine models, the inflammatory process occurred before fibrosis formation. The RNA-Seq results indicate that the conversion from inflammation to fibrosis was largely modulated by neutrophil activation. Intriguingly, among those DEGs involved in neutrophil degranulation, several have already been identified in the development of fibrosis. For example, Ptafr has been found to be significantly dysregulated in the liver (Cai et al., 2006) and cardiac fibrosis (Zhao et al., 2020b). Liang et al. demonstrated that inhibiting Ptafr could block the cell proliferation and secretion of collagen (Liang et al., 2018). Of note, Sala et al. also identified differentially expressed Ptafr in the nasal epithelium of IPF patients (Sala et al., 2018). All these findings of dysregulated Ptafr in animal fibrosis models or in patients could be used to establish the potential role of Ptafr in the development of fibrosis. In addition to elevated neutrophil degranulation, the immune response and chemokine levels were also markedly activated during the inflammatory process induced by bleomycin. A large number of these DEGs consisted of the CXC chemokine family. The CC chemokine family and CC chemokines that bind to CC receptor (CCR) family members do not play a critical role in the recruitment of leukocytes to local inflammation but are important in integrating cellular signal transduction (Starr et al., 2012). For cellular components, DEGs involved in the regulation of the extracellular space were significantly expressed. Some DEGs, such as Tnf, Col3a1, Mmp12, Serpina10, Cd109, Col8a1, Il6, Wnt7b, Fbn1, and Plau, were significantly upregulated in the extracellular space component. Intriguingly, DEGs such as Tff2, Fabp3, Adipoq, Azgp1, Scube2, Cfd, Ces1e, Pgc, Gkn3, Igfbp3, Rnase4, Sema4 g, Nrg4, and S100a7a were downregulated. These findings indicated that the extracellular space changed markedly after intratracheal instillation of bleomycin, and this result provided a molecular basis for bleomycin-induced pulmonary fibrosis *in vitro*. For annotations of molecular functions, DEGs were mainly enriched in the regulation of cytokine activity and binding, such as in CXC chemokines; CC chemokines and CCR family members were markedly upregulated, and calcium ion binding, troponin C binding, and haptoglobin binding were significantly downregulated. In terms of the role of these differentially expressed chemokines involved in the upregulation of related biological processes and molecular functions, we can infer that bleomycin had a great effect on the expression of chemokines and may enhance fibrosis through these chemokines.

Given that pulmonary fibrosis is a progressive fatal disease and that this pathological process can be mimicked in mice by bleomycin to some extent, dynamic gene pattern expression analyses are also needed to further study the disease and explore possible therapeutic targets.

STEM can reflect the gene expression trend at different times (Ernst and Bar-Joseph, 2006). Through the STEM analysis, two profiles caught our attention: Profile 39, which was a consistent upregulation profile, and Profile 8, which was a continuous downregulation profile. GO annotations revealed that the critical functions of the genes in these profiles were involved in three aspects. For Profile 39, in addition to collagen synthesis, extracellular space–related functions and inflammation-related responses were enriched, which have been well studied as a result of intratracheal instillation of bleomycin, and other annotations were also continually significantly upregulated, such as peptidyl–lysine oxidation. Previous research has reported that lysyl oxidase exerts a central role in collagen maturation (HM, 2000). Aumiller et al. demonstrated that Loxl2 is strongly expressed in human lung fibroblasts and airway epithelial cells after stimulation with TGF-β. In addition, they also found increased expression of Loxl2, Loxl3, and Loxl4 in whole lung tissue from pulmonary fibrosis mouse models using gene expression assays at day 14 post-bleomycin. Consistent with these findings, the expression of Loxl2, Loxl3, and Loxl4 was also increased in the tissue of IPF patients (Aumiller et al., 2017). Similarly, Tjin et al. found that Loxl2 was upregulated in IPF patients (Tjin et al., 2017). Consistent with these results, Loxl2, Loxl3, and Loxl4 were continually upregulated in our research (Supplementary Excel 2). Additionally, Nuggc was also found to be continually increased during the regulation of peptidyl−lysine oxidation. However, to the best of our knowledge, Nuggc has not been previously reported in the regulation of the development of fibrosis and deserves further research.

For the continually decreased Profile 8, annotations were mainly enriched in cardiac functions, such as cardiac muscle organization and contraction. In IPF patients, cardiovascular comorbidities, such as hypertension, coronary heart disease, and heart failure, represent important factors that affect the prognosis and mortality of IPF (Agrawal et al., 2016). Research is limited on the relationship between cardiac muscle function and pulmonary fibrosis. In the current research, sarcomere organization, muscle contraction, and muscle fiber development were continually downregulated post-bleomycin. Related genes, such as Tnnt2, Actc1, and Myh6, were implicated. However, dysregulation of these genes may be the leading factor for the continually decreased cardiac functions and could act as a stimulus for the severity of the disease. For example, mutations of Tnnt2 lead to the loss of sarcomeres and cause the disarrangement of myocytes, which can be a very important life-threatening hallmark for patients (Sehnert et al., 2002). Actc1 is reduced in patients with congenital heart disease, while its downregulation could be responsible for cardiomyocyte apoptosis (Jiang et al., 2010). Downregulation of Myh6 occurred concomitantly with a reduced ejection fraction of systemic right ventricular tissue in patients with hypoplastic left heart syndrome (Theis et al., 2015). In the current research, continually downregulated genes enriched in cardiac functions may be responsible for the poor prognosis and heart complications resulting from the pulmonary fibrosis, which also provides a molecular mechanism insight into the cardiac complications that result from the pulmonary fibrosis. However, whether these continually downregulated genes are directly involved in the regulation of pulmonary fibrosis remains unclear, and further research exploring the mechanism underlying their dysregulation in the context of pulmonary fibrosis is still essential.

In addition, we also explored the KEGG pathways enriched in Profile 39 and Profile 8. For Profile 39, the PI3K-Akt signaling, ECM–receptor interaction, and focal adhesion signaling pathways were continually upregulated. PI3K-Akt plays a critical role in a wide range of cellular processes, such as cell metabolism, proliferation, growth, and protein synthesis (Kuma et al., 2004). Previous research by Lu et al. found that aberrant activation of PI3K-Akt promotes fibroblast proliferation and collagen synthesis (Lu et al., 2010). Similarly, Zhang et al. also demonstrated that PI3K-Akt is involved in the development of pulmonary fibrosis using a bleomycin-induced rat pulmonary fibrosis model, while inhibition of PI3K-Akt could reverse pulmonary fibrosis and epithelial mesenchymal transition (Zhang et al., 2016). In addition, PI3K-Akt is also a bridge that communicates signals to various pathways, and it also plays an important role in pulmonary fibrosis, such as mTOR, VEGF, and TGF-β (Yan et al., 2014). Given the roles of PI3K-Akt in pulmonary fibrosis, research has shown that the PI3K-Akt signaling pathway could be a therapeutic target in IPF patients. Accordingly, a study reported that omipalisib (GSK2126458), an inhibitor of PI3K-mTOR, was under Phase I clinical trial in the treatment of IPF (Lukey et al., 2019). Compared with these studies, continually upregulated PI3K-Akt was found in our research based on a STEM analysis, and it was enriched along with genes that were previously reported in the context of fibrosis, such as collagen1, collagen4, Il7r, Itga11, and Itgb6. Our research is consistent with previous findings, suggesting a critical role of these genes in the upregulation of the PI3K-Akt signaling pathway throughout the development of pulmonary fibrosis. Of note, several genes, such as Reln and Ntrk2, were also enriched in PI3K-Akt. Reln was reported to be related to liver fibrosis (Mansy et al., 2014), and Ntrk2 encodes a tyrosine kinase receptor, TrkB, which is abundantly expressed in lung tissue and responsible for hypoxia, thus promoting the proliferation of pulmonary arterial smooth muscle cells (Kwapiszewska et al., 2012). These results and our findings could provide new insights into the genes involved in pulmonary fibrosis. However, further research is still essential.

For the KEGG analysis of Profile 8, continually downregulated pathways were the cAMP signaling pathway, the cGMP-PKG signaling pathway, the calcium signaling pathway, and cardiac muscle contraction. Enriched genes, such as Actc1, Adrb2, Adcy8, and Chrm1, may correspond to some critical functions. For example, Adrb2 controls the expression of the β2 adrenergic receptor, which belongs to the G-protein-coupled family and plays a critical role in regulating the pulmonary, cardiac, and vascular systems by binding to catecholamines and epinephrine (Litonjua et al., 2010). Adcy8 is known to regulate glucose homeostasis (Raoux et al., 2015). Chrm1 belongs to the muscarinic–cholinergic receptor (CHRM) family and encodes the Chrm1 receptor, which is extensively expressed in the lung and regulates the secretion of electrolytes and water (Jimenez-Morales et al., 2014). Their continuous downregulation may also provide therapeutic insights into new targets for the treatment of pulmonary fibrosis.

However, this research presented certain limitations. We monitored autophagy markers in the whole lung tissue, and it seems that autophagy did not exert a critical role in a continually changing manner, whereas it might play an important role at a certain time in a certain cell type which deserves further study. Given that epithelial cells and fibroblasts are the two main cell types involving pulmonary fibrosis, either monitoring the expression of autophagy-related gene expression or observing the specific structure such as autophagosomes in certain cell types besides these could be meaningful. Furthermore, in the current study we have assessed autophagy in the whole development of fibrosis. It is better to use the autophagic inducer or inhibitor to assess the dynamic change of autophagic flux and further, to evaluate the role of autophagy in the alleviation or aggregation of fibrosis. Also, a specific autophagy-deficient or aberrantly activated animal model for studying pulmonary fibrosis could be more indicative for the related research. Additionally, although bleomycin is widely used for establishing pulmonary fibrosis models, these models are not consistent with those for IPF patients. IPF is a progressive and irreversible disease, but the process is difficult to reproduce completely in a mouse model induced by intratracheal instillation of bleomycin. In the pathology of mouse fibrosis, as the disease progresses, lung pathology tends to recover 28 days after bleomycin treatment. Although many DEGs and pathways were identified by RNA-Seq, it is still difficult to determine the most important genes in the development of IPF since the disease was thought to be a result of multiple factor interactions. The results would be more robust if those genes or pathways were further validated in the patients’ tissue.

## Conclusion

In the current research, we monitored autophagy at different stages of pulmonary fibrosis using bleomycin-induced mouse models. We detect autophagy markers through multiple aspects. Additionally, we analyzed autophagy-related genes from public datasets. All results consistently indicate that autophagy did not exhibit a continual expression in the development of fibrosis. We then explored the associated DEGs, including their enriched functions and signaling pathways, based on RNA-Seq. Continually upregulated annotations were related to collagen synthesis, inflammation, and extracellular space. The enriched signaling pathways were mainly related to the PI3K-Akt signaling, ECM–receptor interaction, and focal adhesion signaling pathways. Continually downregulated profiles were mainly related to sarcomere organization, muscle contraction, and muscle fiber development. Enriched KEGG signaling pathways were related to the cAMP signaling, cGMP-PKG signaling, calcium signaling, and cardiac muscle contraction pathways. In summary, autophagy may not be critically involved in the development of pulmonary fibrosis. These continually up- or downregulated profiles, including the associated genes and their functions and pathways, may provide mechanistic insights for future therapies for pulmonary fibrosis.

## Data Availability

The datasets presented in this study can be found in online repositories. The names of the repository/repositories and accession number(s) can be found below: NCBI SRA Database Repository, accession number PRJNA726799.
