# Supplementary material for: Dynamic Observation of Autophagy and Transcriptome Profiles in a Mouse Model of Bleomycin-Induced Pulmonary Fibrosis
Source: Front Mol Biosci. 2021 Jul 29;8:664913. doi: 10.3389/fmolb.2021.664913 (PMC8358296; doi:10.3389/fmolb.2021.664913)
Supplement: Supplementary file 6 [file DataSheet1.docx]

**Supplementary File**

**Dynamic observation of autophagy and transcriptome profiles in a mouse model of bleomycin-induced pulmonary fibrosis**

Yani Wang^1^, Siqi Hu^1,2^, Lisha Shen^1,3^, Song Liu^4^, Linyan Wan^5^, Shuhui Yang^5^, Mengjie Hou^5^, Xinlun Tian^1^, Hongbing Zhang^5^, Kai-Feng Xu^1*^

^1^Department of Pulmonary and Critical Care Medicine, State Key Laboratory of Complex Severe and Rare Diseases, Peking Union Medical College Hospital, Chinese Academy of Medical Sciences, Beijing, China.

^2^Department of Pulmonary and Critical Care Medicine, Subei People’s Hospital of Jiangsu Province, Yangzhou, China.

^3^Department of Pulmonary and Critical Care Medicine, The First Affiliated Hospital of Zhejiang University School of Medicine, Hangzhou, China.

^4^Medical Science Center, State Key Laboratory of Complex Severe and Rare Diseases, Peking Union Medical College Hospital, Chinese Academy of Medical Sciences, Beijing, China.

^5^Department of Physiology, Institutes of Basic Medical Sciences, Peking Union Medical College, Chinese Academy of Medical Sciences, Beijing, China.

* Corresponding author: xukf@pumch.cn

**Material and methods**

**Reagents and materials**

Bleomycin was purchased from Nippon Kayaku Co., Ltd (Tokyo, Japan). SQSTM1/p62 rabbit antibody (Cat. No. 23214, 1:1000), α-SMA rabbit antibody (Cat. No. 19245, 1:1000) and β-actin rabbit antibody (Cat. No. 4967, 1:1000) were from Cell Signaling Technology (Danvers, MA). LC3B rabbit polyclonal antibody (Cat. No. ab48394, 1:1000), Atg4b rabbit monoclonal antibody (Cat. No. ab154843, 1:1000), Atg16L1 rabbit monoclonal antibody (Cat. No. ab187671, 1:1000) and Atg5 rabbit monoclonal antibody (Cat. No. ab108327, 1:1000) were bought from Abcam (Cambridge, MA). HRP-labeled secondary antibodies were purchased from Santa Cruz Biotechnology (Santa Cruz, CA). 4-12% NuPAGE Bis-Tris Gel was supplied by Life Technologies (Carlsbad, CA).

**Mice** **and bleomycin treatment**

C57BL/6J male mice were purchased from Vital River Lab Animal Technology (Beijing, China). Mice were maintained and treated at the Institute of Laboratory Animal Sciences, Chinese Academy of Medical Sciences and Peking Union Medical College (CAMS/PUMC) in accordance with the guidelines strictly. All mice experimental procedures were approved by the Animal Ethics Committee of Peking Union Medical College (NO. XHDW-2017-010).

For bleomycin induced pulmonary fibrosis, 6-8 weeks old mice weighted 20-25g were anesthetized with 0.6% sodium pentobarbital followed by intratracheal injections of bleomycin (2.5 mg/kg) or sterile saline as previously described (14). Mice were sacrificed at the day of 3, 7, 14, 21, 28 after intratracheal injection with bleomycin or saline. Lung tissue were collected after perfused with sterile saline from right ventricle.

**Bronchoalveolar lavage fluid (BALF)**

The BALF was collected in each group of mouse post-bleomycin at the indicated day. Mouse lung was lavaged using 0.4 ml phosphate buffer saline (PBS) each time and repeatedly for three times. The collected total BALF was 0.5ml or more. Then the BALF was centrifuged at 1000 rpm, at 4℃ for 10 min. The pellet was resuspended with PBS followed by removing the red blood cells using erythrocyte lysate (Solarbio, Beijing, China) at room temperature for 10 min. The total viable cell numbers were counted with a cell counter (Luna Ⅱ, Logos Biosystem, Korea) according to the manufacturer’s instruction.

**Histology analysis**

Lung tissue were removed and collected carefully after rinsed with PBS for several times. Then fixed in a container with 4% paraformaldehyde overnight. After embedded in paraffin, a 5μm thickness section slices were obtained followed by hematoxylin-eosin (H&E) staining and Masson trichrome staining. The pulmonary fibrosis extent was evaluated under an inverted phase contrast microscope (Nikon, Japan).

**Immunohistochemistry analysis**

5μm thickness section slices of lung tissue embedded in paraffin were deparaffinized and rehydrated in xylene. Then antigen retrieval was conducted using 10 mM sodium heated in a microwave oven. Tissue sections were treated with 3% H_2_O_2_ for 10min to cease the endogenous peroxidase activity. After blocked with 5% normal goat serum for 2h, tissue sections were incubated with the primary antibody against α-SMA at 4°C overnight. The secondary antibody was used at room temperature for 1h followed by the treatment of diaminobenzidine (DAB) regent according to the manufacturer’s instructions (Absin, Shanghai, China). Finally, tissue sections were observed and imaged under an optical microscope (Nikon, Japan).

**Immunofluorescence staining**

5μm thickness section slices of lung tissue were dewaxed and rehydrated followed by blocking and incubated with LC3B rabbit polyclonal antibody at 4 ℃ overnight. Alexa Fluor-488-conjugated goat anti-rabbit IgG H&L secondary antibody (abcam, Cat No. ab150077,1:1000) were used for visualization. The slides were then rinsed and counterstained with DAPI. Finally, the sections were observed by a confocal microscope (Olympus FV1000, Tokyo, Japan).

**Transmission electron microscopy (TEM) analysis**

Mice were sacrificed at the indicated days after intratracheal injection with bleomycin or the sterile saline. Left lungs of mice were collected after fixed with 2.5% glutaraldehyde for 2h followed by fixation in 1% osmium tetroxide. Then tissue was dehydrated with ethanol and treated with propylene oxide followed by embedded in epoxy resin. 50-70 nm thickness sections were prepared for observation under a transmission electron microscopy (JEM-1400 Plus, JEOL Ltd, Japan).

**Immunoblotting**

Total tissue protein was extracted using RIPA lysate (Beyotime Biotechnology, Haimen, China). The protein expression was measured as previously described (15, 16). In brief, mice lung tissue was collected after perfused with sterile saline from right ventricle and rinsed in PBS buffer. The tissue lysates were subjected into 4-12% NuPAGE Bis-Tris gels for electrophoresis followed by transferred to a PVDF membrane (Millipore, MA, USA). The membrane was blocked in 5% nonfat milk (BBI Life Sciences, Shanghai, China) and incubated with the primary antibodies at 4°C overnight. After washed by tris-buffered saline with 0.1% Tween 20 and incubated with secondary antibodies for an hour at room temperature, protein immunostaining was performed and imaged using an enhanced chemiluminescence kit according to the manufacturer’s instructions (Thermo Scientific).

**Quantitative RT-PCR (qRT-PCR)**

Total lung tissue RNA was extracted using the TRIzol (Life Technologies, MA, USA) regent according to the manufacturer’s instructions. 1μg RNA was used as the template to synthesis the first-strand cDNA using a PrimeScript^TM^ RT Master Mix Kit (Takara, Shiga, Japan). Then cDNA was diluted for 20 times as the template for real time PCR using a TB Green Premix Ex Taq^TM^ II Kit (TaKaRa, Shiga, Japan). Total reaction system was 20 μl and genes amplification process was performed via Applied Biosystems 7500 Fast Real-Time PCR System (Life Technologies). The RNA expression of target genes was calculated using 2^−ΔΔCt^ method on the basis of housekeep gene β-Actin. Every sample was performed three times with triplicates. The primers of target genes were listed in Table. S1

**Table S1. Primers used in qRT-PCR**

| **Genes** | **Primer Sequence (5’-3’)** |
| --- | --- |
| Reln | Forward 5’-TTACTCGCACCTTGCTGAAAT-3’  Reverse 5’-CAGTTGCTGGTAGGAGTCAAAG-3’ |
| Igf1 | Forward 5’-CTGGACCAGAGACCCTTTGC-3’  Reverse 5’-GGACGGGGACTTCTGAGTCTT-3’ |
| Col1a1 | Forward 5’-GCTCCTCTTAGGGGCCACT-3’  Reverse 5’-CCACGTCTCACCATTGGGG-3’ |
| Actc1 | Forward 5’-CTGGATTCTGGCGATGGTGTA-3’  Reverse 5’-CGGACAATTTCACGTTCAGCA-3’ |
| Cst8 | Forward 5’-GTGTTTGGTTTGCCATGAAAGAA-3’  Reverse 5’-TGGTATTCCATTCGGTCTGTGAT-3’ |
| Myh6 | Forward 5’-GCCCAGTACCTCCGAAAGTC-3’  Reverse 5’-GCCTTAACATACTCCTCCTTGTC-3’ |
| β-Actin | Forward 5’-GGCTGTATTCCCCTCCATCG-3’  Reverse 5’-CCAGTTGGTAACAATGCCATGT-3’ |

**Results**

**Development of pulmonary fibrosis of mouse model in different stages post-bleomycin**

Given that the autophagy was a dynamic process which often influenced by many factors. While pathogenesis of pulmonary fibrosis mouse model induced by intratracheal injection of bleomycin was based on the initiation of inflammatory process, followed by transition to the fibrosis after 7-14 days. Hence, we divided the mouse into the group of day 3, 7, 14, 21 and day 28 post-bleomycin, respectively, to observe the development of fibrosis in lung. As depicted in Fig. S1a, the total viable cell counts in mouse instilled with bleomycin showed more cells in bronchoalveolar lavage fluid (BALF) than the control, especially between the day 7 to day 14. While the pulmonary fibrosis in mouse treated with bleomycin appeared progressively worsened concomitantly with accumulative of inflammatory cell infiltration, particularly during the day 14 to day 28 (Fig. S1b). We then evaluated the collagen deposition by Masson staining and fibrosis scoring. In comparison with the control, mouse treated with bleomycin showed severer deposition of collagen. The structure of alveolar was markedly distorted with much deposition of extracellular matrix (Fig. S1c-d). Additionally, we also explored the expression of α-SMA, the hallmark of myofibroblasts. In consistent with the results from H&E and Masson staining, expression of α-SMA were increased, much obvious between the day 14 to day 28 indicating an accumulated myofibroblast foci deposited in the lung interstitium (Fig. S1e). Taken together, current research shows that in pulmonary fibrosis mouse model induced by intratracheal instillation of bleomycin, inflammation peaked from day 7 to day 14 followed by the alleviation of inflammation and concomitantly gradual severer pulmonary fibrosis developed during the day 14 to day 28 post bleomycin.

**
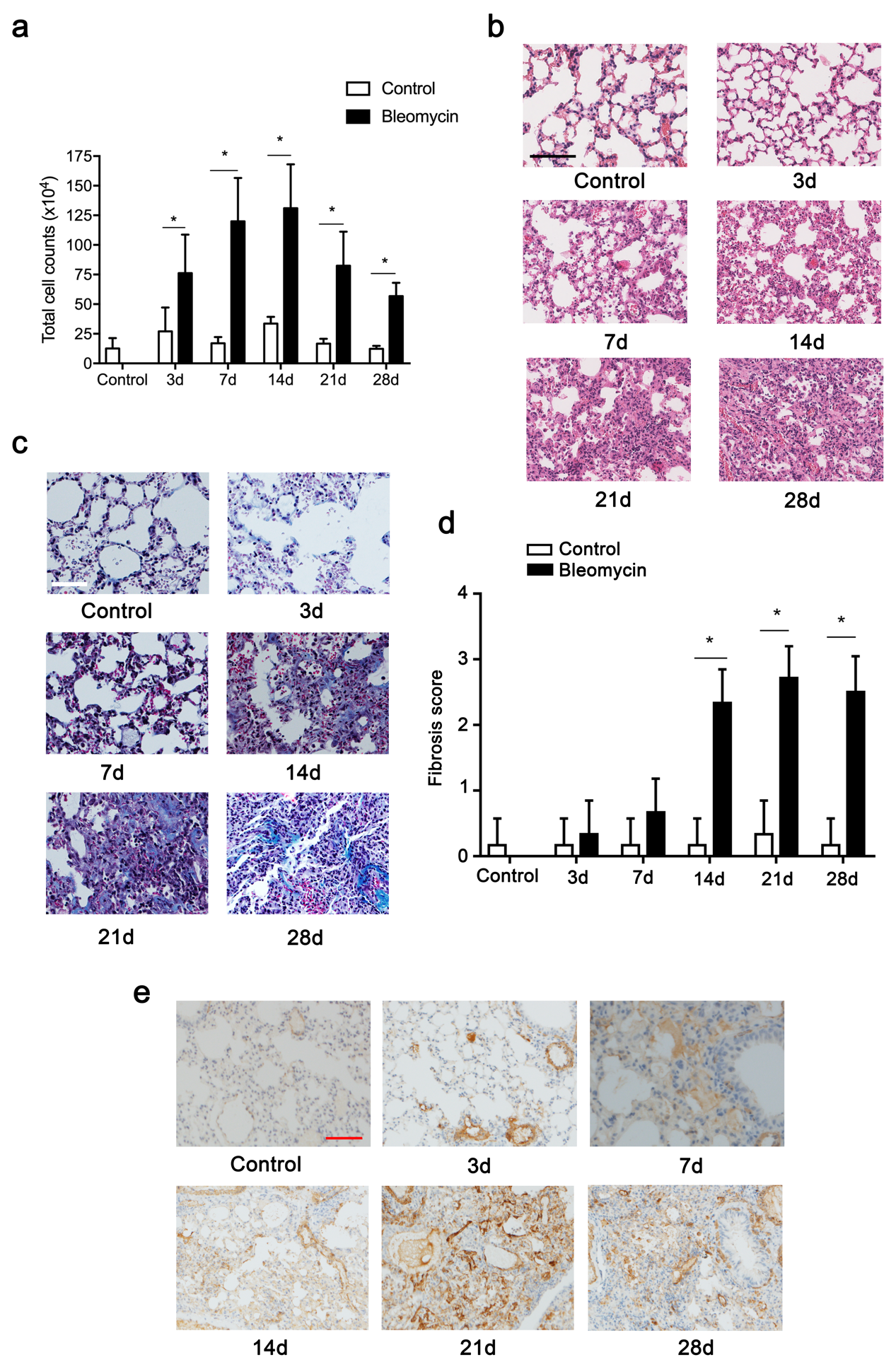
**

**Fig. S1 Development of lung fibrosis at different stages post bleomycin.**

a. Total viable cell counts in bronchoalveolar lavage fluid (BALF) from mice with intratracheal instillation of bleomycin at indicated days. b. H&E staining of lung tissue of mice at indicated days post-bleomycin. Scale bar: 100 μm. c. Masson trichrome staining of lung tissue of mice at indicated days after intratracheal instillation of bleomycin. Scale bar: 50 μm. d. Fibrosis score of lung tissue at different stages post-bleomycin. e. Representative images of deposition of α-SMA of mouse lung tissue at indicated days post-bleomycin. Scale bar: 50 μm. Error bars indicate mean ± SD of triplicate samples. **p*< 0.05.

**Quality analysis of samples for RNA-Seq**

To determine the differentially expressed genes and their functions, as well as the pathways they involved in during the development of pulmonary fibrosis in different stages of mouse model post-bleomycin, RNA-Seq was performed. Firstly, principal component analysis (PCA) was used to identify the distribution and the correlation between the samples during different stages post-bleomycin. As depicted in Fig. S2, the control samples and bleomycin treated samples were divided into two clusters. PC score plots showed that 45.98%, 20.4% and 9.83% variance in three principal components, PC1, PC2 and PC3 respectively. In addition, though there are variations among samples, no outlier was appeared, which indicated that the data were eligible for the following analysis.

**Fig. S2 Quality analysis of samples for RNA-Seq**
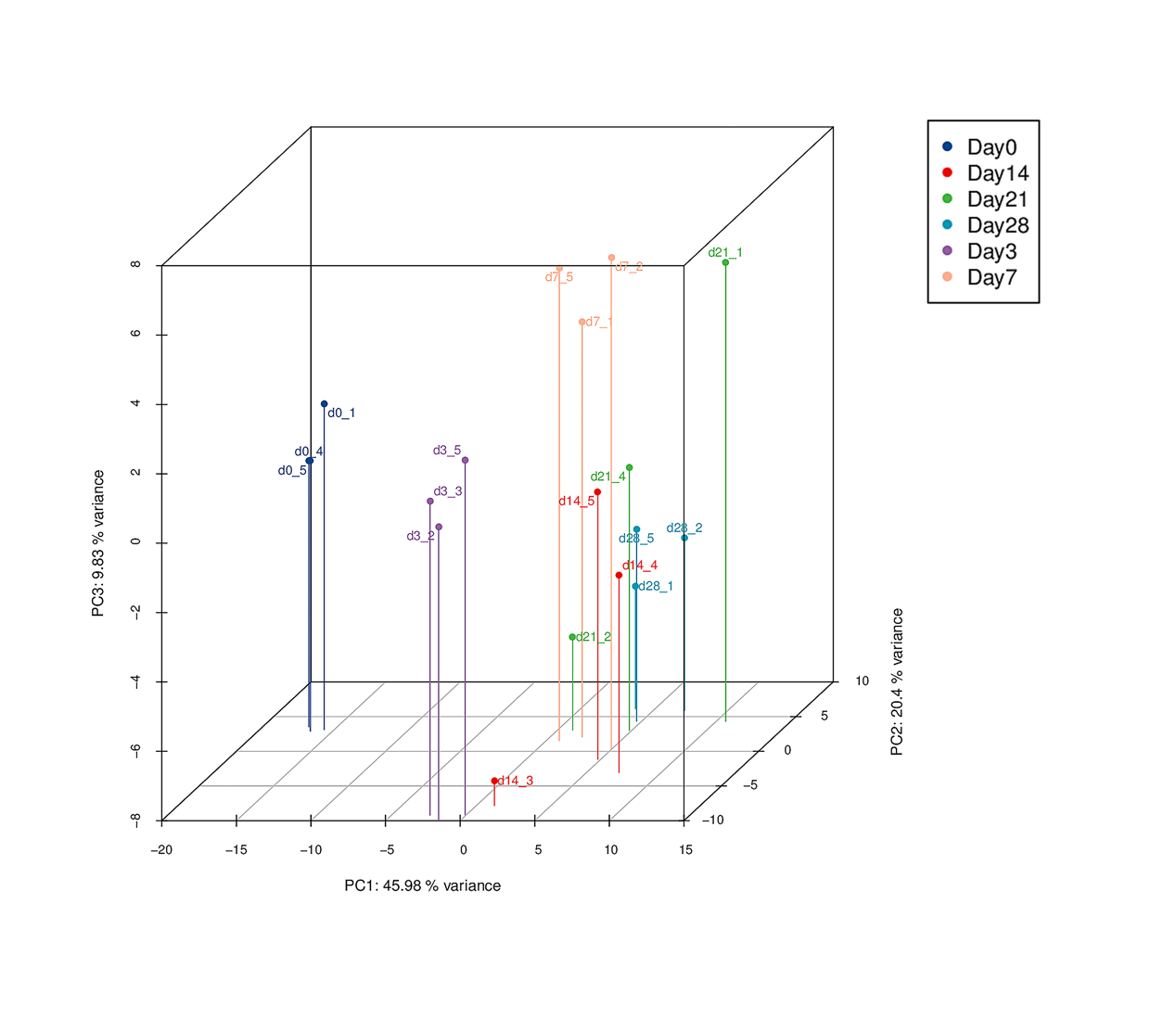


Principal component analysis (PCA) shows the distribution of samples at indicated days post-bleomycin. PC score plots explain that 45.98%, 20.4% and 9.83% variance in PC1, PC2 and PC3 respectively.

**Differentially expressed genes in pulmonary fibrosis at different stages post-bleomycin**

The cutoff value for differentially expressed genes (DEGs) was defined as *P* value < 0.05 and foldchange >2 or foldchange < 0.5. In comparison with the control, we have identified a total of 895 upregulated DEGs and 413 downregulated DEGs at day 3 post-bleomycin; 1114 upregulated and 862 downregulated DEGs at day 7; 1007 upregulated and 590 downregulated DEGs at day 14; 978 upregulated and 616 downregulated DEGs at day 21; and 1185 upregulated and 685 downregulated DEGs at day 28 (Supplementary Excel 1). The whole numbers of DEGs between the groups, as well as the numbers of common DEGs they shared were showed in Fig. S3a-b.

To explore the DEGs between the groups of the mice treated with bleomycin and the control, as well as the expression of DEGs in different stages post-bleomycin, cluster analysis of DEGs was performed. As depicted in Fig. S3c, the volcano map has described the distribution of DEGs and also reflect the relationship between the genes and statistical significance. While hierarchical cluster analysis has revealed the relationship between genes expression patterns and samples (Fig. S3d). Samples with similar patterns and genes with the same biological function were gathered to the same clusters. Samples collected at the same time were gathered into the same side and the control samples were gathered into the same clusters, which also indicate that the samples were eligible for analysis and no outlier was shown. Taken together, the data indicate that a great deal of genes was significantly changed in the pathogenesis of pulmonary fibrosis mouse models induced by bleomycin.


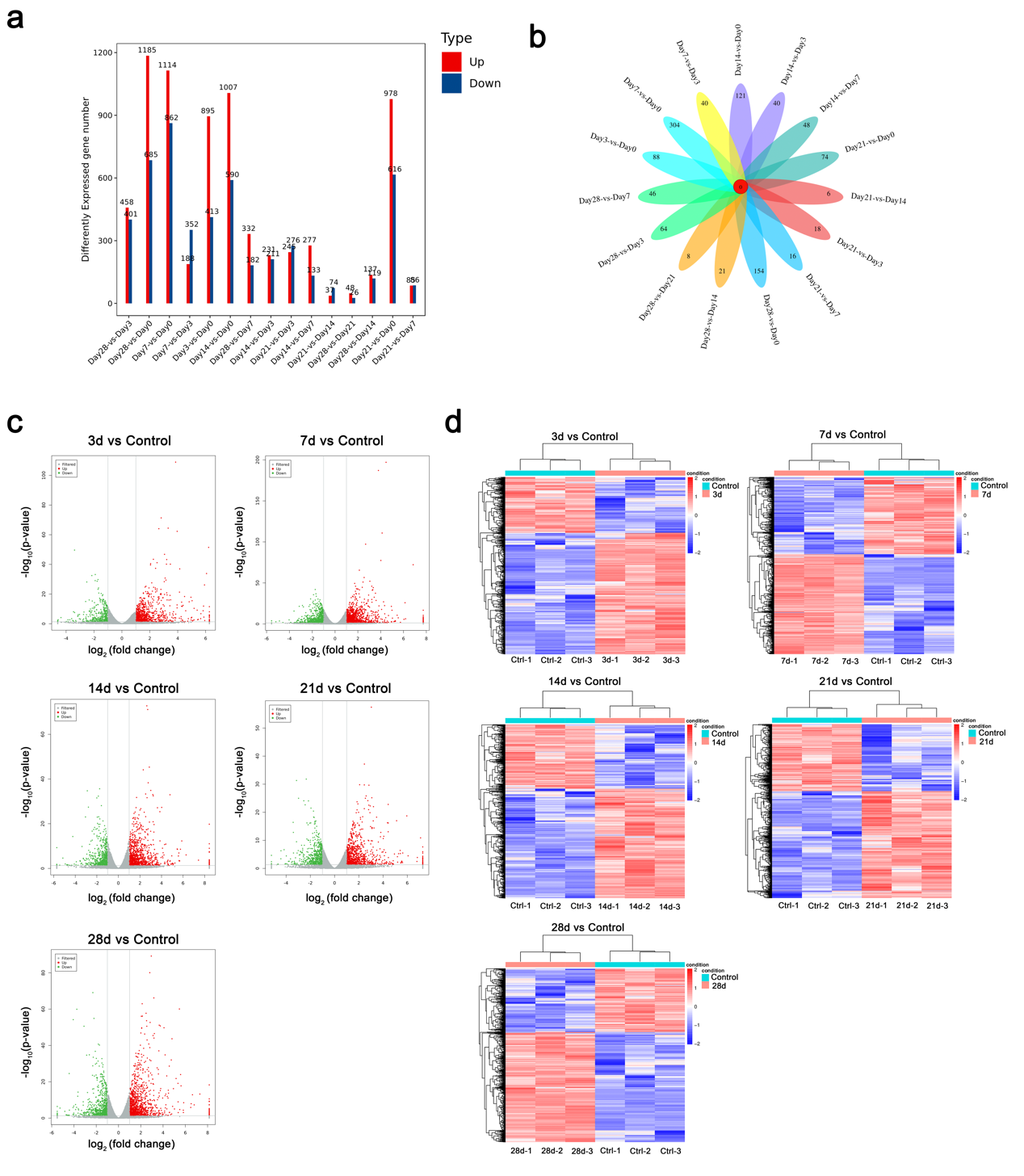


**Fig. S3 Differentially expressed genes at different stages post-bleomycin treatment**

a. Numbers of differentially expressed genes between the groups. Red means upregulated differentially expressed genes and blue represents downregulated genes. b. The numbers of common and unique differentially expressed genes between the groups as indicated. c. Distribution of differentially expressed genes between the group of mice post-bleomycin at indicated day and the control in volcano map. Red: upregulation; green: downregulation; gray: no statistical difference. d. Hierarchical cluster analysis of differentially expressed genes between the group of mice post-bleomycin at indicated day and the control. Red: upregulation. Blue: downregulation. Cutoff value for differentially expressed genes was defied as P value < 0.05 and foldchange >2 or foldchange < 0.5.
